# A New Species and Molecular Characterization of the Genus *Phraepsyche* Malicky and Chantaramongkol, 2000, from China (Trichoptera, Odontoceridae)

**DOI:** 10.3390/ani16142209

**Published:** 2026-07-16

**Authors:** Lu Chai, Ni Zhang, Na Wang, Xinyu Ge, Zheng Liu

**Affiliations:** 1 Tianjin Key Laboratory of Conservation and Utilization of Animal Diversity, College of Life Sciences, Tianjin Normal University, Tianjin 300387, China; 2410170016@stu.tjnu.edu.cn (L.C.); 2510170030@stu.tjnu.edu.cn (N.Z.); 2510170025@stu.tjnu.edu.cn (N.W.); 2 Geological Museum of China, Beijing 100034, China

**Keywords:** taxonomy, phylogeny, mitogenome, Oriental region

## Abstract

The genus *Phraepsyche* Malicky and Chantaramongkol, 2000, is endemic to the Oriental region, currently comprising five species. However, its species diversity remains incompletely explored, and genetic data for this genus are completely lacking. Here, we describe a new species of *Phraepsyche* from southwestern China, present a distribution map of the species in the genus, and update the key to *Phraepsyche*. Meanwhile, we obtained the first genetic data (mitochondrial genome) for *Phraepsyche* and performed assembly, annotation, and structural analysis of the mitogenome. Furthermore, we inferred the phylogenetic position of *Phraepsyche* using representatives of other genera of Odontoceridae.

## Simple Summary

The genus *Phraepsyche* is distributed in the Oriental region; however, its species diversity remains poorly studied, and no genetic data have been available. In this study, we report a new species of *Phraepsyche* from southwestern China. We also present the first mitochondrial genome for this genus and use it to infer the phylogenetic position of *Phraepsyche* within the related family Odontoceridae. This research enhances our understanding of freshwater insect biodiversity in China and provides valuable genetic resources for future conservation and evolutionary studies.

## 1. Introduction

The family Odontoceridae is a widely distributed yet species-poor family within the suborder Integripalpia. It is divided into two subfamilies: Odontocerinae (14 genera) and Pseudogoerinae (monotypic, containing only *Pseudogoera* Carpenter, 1933). Currently, 177 species in 15 genera are recognized worldwide, four of which are monotypic: *Namamyia* Banks, 1905; *Nerophilus* Banks, 1899; *Pseudogoera* Carpenter, 1933; and *Anastomoneura* Huamantinco and Nessimian, 2004 [1]. Most genera in this family have restricted, regionally endemic distributions, with the highest species diversity concentrated in the Oriental and Neotropical regions [2]. In China, 45 odontocerid species belonging to four genera have been recorded to date: two species of *Lannapsyche*, seven species of *Marilia*, 34 species of *Psilotreta*, and two species of *Phraepsyche* [3].

The genus *Phraepsyche* was erected by Malicky & Chantaramongkol [4], with *Phraepsyche danaos* designated as the type species. Subsequent taxonomic studies have steadily expanded its known species diversity [4,5,6]. Currently, five species are recognized in this exclusively Oriental genus: *P. danaos* Malicky and Chantaramongkol, 2000 (Thailand), *P. epha* Malicky, 2008 (Vietnam), *P. pectinata* Oláh and Johanson, 2010 (Vietnam), *P. yitungshana* Oláh and Johanson, 2010 (Hong Kong, China), and *P. acuminata* Yang and Morse, 2020 (Guangxi, China) [3].

The mitochondrial genome (mitogenome) is maternally inherited and exists as a closed circular double-stranded DNA molecule independent of the nuclear genome. In insects, mitogenomes typically range from 15 to 18 kb in length and encode a highly conserved set of 37 genes, including 13 protein-coding genes (PCGs), 22 transfer RNA (tRNA) genes, two ribosomal RNA (rRNA) genes, and a non-coding AT-rich control region [7]. With a moderate size, high copy number, and abundant genetic information, it can effectively distinguish closely related species [8]. Within the order Trichoptera, mitogenomic data play an irreplaceable role across multiple core research domains, including phylogenetic reconstruction, population evolutionary biology, species delimitation, and aquatic ecological monitoring [9,10]. As “DNA Barcoding 2.0”, the mitogenome has become an important tool for species classification and eDNA metabarcoding studies [11,12]. Compared with the standard COI barcode fragment, whole mitogenomes provide substantially more nucleotide variation and multiple independent genetic loci, enabling more reliable species delimitation and finer phylogenetic resolution. For eDNA-based freshwater biodiversity surveys, high-quality mitogenome reference databases can significantly improve taxonomic resolution and reduce false positive detection rates, making them particularly valuable for routine biomonitoring targeting Trichoptera and other benthic macroinvertebrates [13]. However, no genetic data for the genus *Phraepsyche* have been published to date, which severely limits progress in species identification, phylogenetic reconstruction, and larval–adult association studies of this genus.

In this study, we described and illustrated a new species, *Phraepsyche coalitus* Ge **sp. nov.**, provided an updated identification key to all species of *Phraepsyche*, and compiled a comprehensive distribution map for all known species in the genus. Meanwhile, we generated and reported the first complete mitogenome for *Phraepsyche* and performed a comprehensive analysis of its nucleotide composition and relative synonymous codon usage. Combined with mitogenomes of four additional species from Odontoceridae, we reconstructed the phylogenetic relationships of the family using the maximum likelihood (ML) method and discussed the phylogenetic placement of *Phraepsyche* within Odontoceridae. Our findings provide a critical foundation for future evolutionary and taxonomic studies of this genus.

## 2. Materials and Methods

### 2.1. Sample Collection and Specimen Examination

Adult specimens were collected using Malaise traps in Guangxi, China, in 2025. Specimens were sorted in the laboratory and preserved in 85% ethanol at −20 °C. Specimen preparation protocols followed Peng et al. [9]. Male abdomens were dissected and cleared in 10% KOH solution by heating in an 80 °C water bath for approximately 10 min to remove non-chitinous tissues and expose male genitalic structures. Cleared genitalia were rinsed in distilled water and preserved in 85% ethanol. Male genitalia were temporarily mounted in lactic acid, and dorsal, ventral, and lateral views were photographed using a Leica M205 C stereomicroscope (Leica Microsystems GmbH, Wetzlar, Germany) equipped with a digital camera. Captured images were imported into Adobe Photoshop 2025 as templates, and digital illustrations were created using a Gaomon G12 graphics tablet (Guangzhou Gaoman Electronic Technology Co., Ltd., Guangzhou, China) to produce the final plates. After documentation, genitalia were transferred to microvials containing 85% ethanol and pinned beneath their corresponding specimens. All voucher specimens are deposited in the Insect Collection of Tianjin Normal University, Tianjin, China (TJNU).

### 2.2. Terminology and Distribution Map

Morphological terminology follows Yang et al. [6] and Oláh and Johanson [5]. Complete distributional records for all valid species of *Phraepsyche* are presented in Table S1. For localities without original GPS coordinates, approximate latitude and longitude values were assigned based on detailed locality information from the original publications to facilitate the construction of the *Phraepsyche* species distribution map. Species spatial distribution maps were generated using ArcGISv10.6.

### 2.3. DNA Extraction and Sequencing

Total genomic DNA was extracted from three legs from one side of a single adult specimen using the TIANamp Genomic DNA Kit (DP304, Tiangen Biotech, Beijing, China) following the manufacturer’s animal tissue protocol. The *mtCOI* barcoding (658bp) was amplified using Taq PCR Master Mix in a 50 μL reaction volume following the protocol of Ge et al. [14]. The universal primers LCO1490 and HCO2198 [15] were used for amplification, with primer sequences provided in Table S2. Amplified PCR products were verified by 1% agarose gel electrophoresis [16].

Purified PCR products were sent to the Beijing Genomics Institute (BGI, Beijing, China) for Sanger sequencing to generate the 658 bp standard DNA barcode region. For mitogenome sequencing, the same genomic DNA sample was submitted to Novogene Co., Ltd. (Beijing, China) for whole-genome shotgun (WGS) sequencing. The sequencing library was constructed following the manufacturer’s standard protocol. A 350 bp insert paired-end library was constructed and sequenced on the BGI DNBSEQ-T7 platform, generating approximately 6 Gb of 150 bp PE raw reads. Raw reads were quality-trimmed using Trimmomatic v0.32 [17], and clean read quality was evaluated with FastQC v0.11.9 [18].

### 2.4. Mitogenome Assembly and Annotation

The raw Sanger sequencing sequences were trimmed, proofread, and assembled using Sequencer v4.5 (Gene Codes Corporation, Ann Arbor, MI, USA) to ensure sequence accuracy. The complete circular mitogenome was assembled de novo using NOVOPlasty v3.8.3 [19] with the *mtCOI* barcoding as the seed sequence and a k-mer size of 39.

The assembled complete mitogenome sequence was annotated to the MITOS2 platform (http://mitos2.bioinf.uni-leipzig.de/index.py accessed on 23 April 2026) to predict 22 tRNAs using the invertebrate mitochondrial genetic code [20]. The annotations of the 13 protein-coding genes (PCGs), two rRNAs, and the control region (CR) of the mitogenome were performed using Geneious Prime 2024.0.2 [21]. As no genetic data were previously available for *Phraepsyche*, homologous sequence alignments of the 13 PCGs and two rRNA genes were conducted individually using Clustal Omega 1.2.2 [22,23], incorporating published mitogenomes of four additional species from other genera of Odontoceridae.

### 2.5. Mitogenome Structure Analysis

For the visualization of the mitogenome structure, the sequence file and annotation information were imported into the Proksee website (https://proksee.ca). GC-skew and GC content were configured, and the platform was used to generate a schematic map of the mitogenome [24]. The schematic map was then refined and beautified using Adobe Illustrator 2022 software (Adobe Inc., San Jose, CA, USA).

Nucleotide composition analysis was performed using SeqKit v2.8.2 [25]. AT-skew and GC-skew values were calculated using the standard formulas: AT-skew = [(A − T)/(A + T)] and GC-skew = [(G − C)/(G + C)] [26]. The relative synonymous codon usage (RSCU) values for the 13 PCGs were calculated using MEGA 12 [27] and R v4.5.2 [28].

### 2.6. Phylogenetic Analysis

To determine the phylogenetic position of *Phraepsyche*, a total of 11 mitogenome sequences from four families (Leptoceridae, Molannidae, Calamoceratidae, and Odontoceridae) of Trichoptera were selected for phylogenetic reconstruction (Table S3). The selection of outgroups followed the phylogenetic analysis of Trichoptera inferred by Ge et al. [29]. All sequences were derived from previously published data in GenBank.

Each PCG and rRNA sequence was aligned using the L-INS-I algorithm in MAFFT v7.490 [30] and trimmed using the “-automated1” strategy in trimAl v1.4.1 [31]. The trimmed alignments were then concatenated into five matrices using FASconCAT-G v1.05 [32]: (1) the CDS_aa matrix, containing the amino acid sequences of all 13 PCGs (parameters: -p -p -s -l); (2) the CDS_123fna matrix, containing the nucleotide sequences of the 13 PCGs (parameters: -p -p -s -l); (3) the CDS_12fna matrix, containing the nucleotide sequences of the first and second codon positions of the 13 PCGs (parameters: -p -p -s -l -d); (4) the CDS_rrna matrix, containing the nucleotide sequences of the 13 PCGs and 2 rRNAs (parameters: -p -p -s -l); (5) and the CDS_12fna+rrna matrix, containing the nucleotide sequences of the first and second codon positions of the 13 PCGs and the 2 rRNAs (parameters: -p -p -s -l -d). The heterogeneity of the five data matrices was calculated using ALIGROOVE v1.0.7 [33], with default parameters to select the optimal matrix for subsequent phylogenetic reconstruction.

The phylogenetic relationship was reconstructed using the maximum likelihood (ML) method. ML analysis was performed using IQ-tree v2.0.7 [34], with ModelFinder [35] used to select the best-fit substitution model for each gene partition. For the amino acid data matrix, the posterior mean site frequency (PMSF) [36] -m mART + C60 + FO + R) mixture model was used for tree reconstruction. Node support for all ML trees was assessed using 1000 ultrafast bootstrap (UFBoot2) replicates [37] and SH-aLRT [38]. The resulting phylogenetic trees were displayed using FigTree v1.4.3 [39].

## 3. Results

### 3.1. Taxonomy

Family Odontoceridae Wallengren, 1891

Subfamily Odontocerinae Wallengren, 1891

Genus *Phraepsyche* Malicky and Chantaramongkol, 2000

Malicky et al. [4] and Oláh and Johanson [5] described the characteristics of the genus as follows: (1) abdominal segment VI with a process bearing setae, extending to segment VII; (2) antennae pectinate; (3) and maxillary palpi 5-segmented; segments 1–3 relatively stout and short, with segments 4 and 5 longer and slenderer. Spur formula 1-4-4.

**Figure 1:**
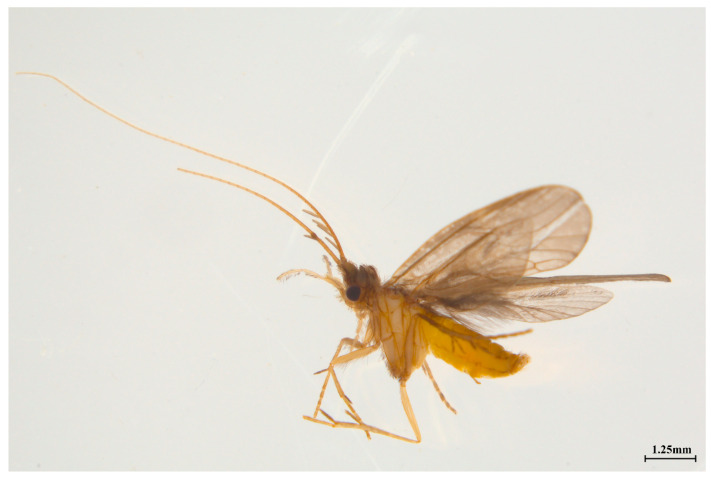
*Phraepsyche coalitus* Ge **sp. nov.**, habitus lateral holotype, male. Scale bars: 1.25 mm.

*Phraepsyche coalitus* Ge **sp. nov.** (Figure 1)

urn:lsid:zoobank.org:pub:402E0370-6E58-4E86-8D39-20135C2B8DB1

Adult. Body length approximately 5.2–5.5 mm, holotype length 5.4 mm (*n* = 4), brown in color. Fore wings broad and rounded apically, with the upturned obtuse apex located between veins R_5_ and MA, a pilose band membrane present, and paler than the rest of the wing. Hind wings narrow and more acute. Spur formula 1-4-4. Maxillary palpi 5-segmented; the fifth segment twice as long as the fourth. Proepisternum with a pair of setal warts, contiguous along the midline. Mesonotum with a pair of separated, long oval setal warts.

**Figure 2:**
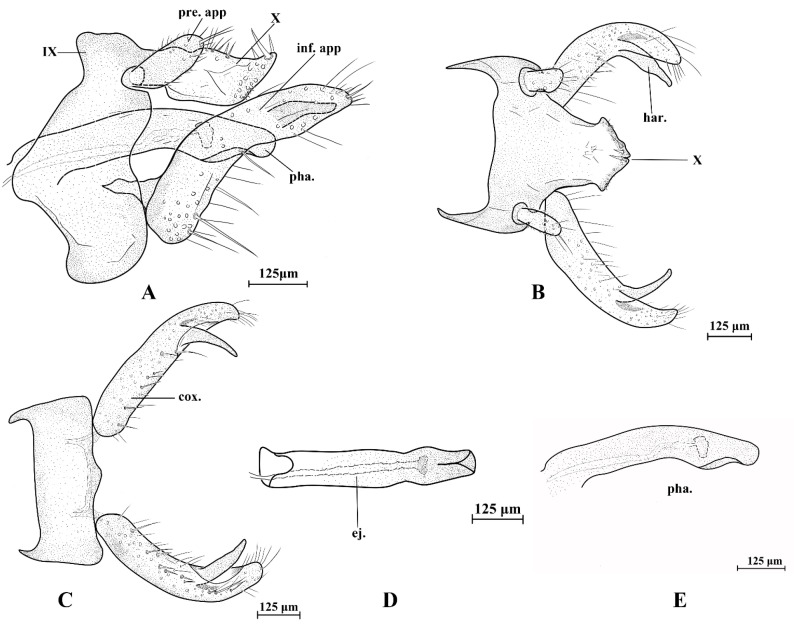
*Phraepsyche coalitus* Ge **sp. nov.**, holotype. (**A**) genitalia, lateral; (**B**) genitalia, dorsal; (**C**) genitalia, ventral; (**D**) phallus, ventral; (**E**) phallus lateral. IX—segment IX; X—segment X; har—harpago of an inferior appendage; cox—coxopodite of an inferior appendage; inf. app—inferior appendage; pre. app—preanal appendage; pha—phallus; ej—ejaculatory duct.

Male genitalia. In lateral view (Figure 2A), maximum longitudinal length of segment IX half height, anterior margins forward process at 1/3 distance from ventral margin. Ventral margin arched and slightly wider than dorsal margin, in dorsal view, with granulose dorsal and lateral surfaces. Tergum X, trapezoidal, ventral margin straight, dorsal margin with setose at middle, distal slightly curving upward in lateral view (Figure 2A). In dorsal view, tergum X with broad base, tapering gradually, distal arrowhead shape triangular, with 5–6 stout setae on distal margin, and middle of distal margin with tiny split (Figure 2B). Preanal appendages in lateral view, rod-shaped, covered with many long and short setae, the shape with narrow base and slightly wider, rounded distal, in lateral view, cylindrical.

Inferior appendages with coxopodite stout, erect, with distal half curved backward (Figure 2A), length between base and distal of appendage about 4 times its width, with many stout setae in ventral view (Figure 2C) and subapicodorsal lobe curving mesad, about as broad as mean width of coxopodite. Harpagones long, goat-horn-like, stout in basal with half distal gradually narrow and curved downward. In ventral view, each arising from distal 1/3 of appendage, with curved inwards distally, robust base and acute distal end, the distal width half of the basal width.

Phallus about as long as inferior appendages in lateral view (Figure 2A), tubular phallus bent downwards at 1/2 length, with surface smooth, the base robust, the distal end thickened then thinned with rounded apex in lateral view. In ventral view, phallus nearly straight (Figure 2D). Ventral margin constricted inwards, narrowed to oval-shaped apex in lateral view. In lateral and ventral views, the ejaculatory duct tubular, lateral margins undulate, ejaculatory duct with conspicuous twist at 1/2 length of phallus.

Diagnosis. This species differs markedly from other members of the genus in the morphology of its genital segments and can be distinguished by the following characteristics: (1) Tergum X of this species gradually fused, while that of other species in the genus comprising a pair of separated triangular lobes (Figure 2B). (2) In lateral view, phallus tubular, bent downwards at 1/2 length, nearly straight in other species of the genus (Figure 2E). (3) In lateral view, tergum X subtrapezoidal, whereas that of other species in the genus subtriangular (Figure 2A).

Type material.

Holotype: ♂; CHINA: Guangxi, Baise City, Napo County; 23°11.85′ N, 105°39.61′ E; alt. 639 m; 1 February 2025–6 March 2025; Malaise trap; Yan-Fei He leg.; [DNA voucher GXBS-051].

Paratypes: 1 ♂; CHINA: Guangxi, Baise City, Napo County; 23°11.85′ N, 105°39.61′ E, alt. 639 m; 24 April 2025–8 May 2025; Malaise trap; Yan-Fei He leg.; 1 ♂; CHINA: Guangxi, Baise City, Napo County; 23°11.20′ N, 105°39.77′ E; alt. 487 m; 21 February 2025–30 March 2025; Malaise trap; Yan-Fei He leg.; 1 ♂; CHINA: Guangxi, Baise City, Napo County; 23°11.85′ N, 105°39.61′ E, alt. 639 m; 6 March 2025–28 March 2025; Malaise trap; Yan-Fei He leg.

Etymology. The species name comes from the Latin adjective coalitus meaning “combined,” or “grown together,” or “united,” in reference to the tergum X fused at the 1/2 distal when viewed dorsally.

**Figure 3:**
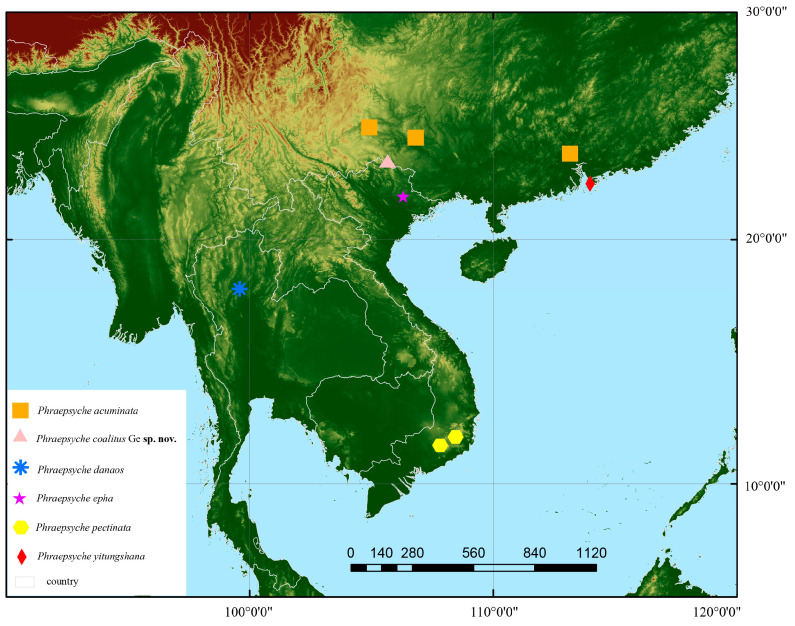
Global distribution of *Phraepsyche* spp.

Distribution. China (Guangxi) (Figure 3).

**Key to species of the genus *Phraepsyche***
1. End of tergum X not divided into two lobes…………………………..……*P. coalitus* Ge **sp. nov.**-End of tergum X divided into two lobes ..………………………….…….…………...………………22. Tergum X expanded laterally before apex ..………………………………………...… *P. danaos* [4]-Tergum X not expanded laterally before apex………………………….………………………….…33. End of inferior appendage acute.…………………………………………………………….………4-End of inferior appendage curved……………………………………….………………….…………54. Harpagones covered with many minute setae……………...…………………………. *P. epha* [40]-Harpagones not covered with many minute setae……………………………..….. *P. acuminata* [6]5. Intersegmental depression between segments IX and X without steps……… *P. yitungshana* [5]-Intersegmental depression between segments IX and X with steps……………..… *P. pectinata* [5]


### 3.2. Mitogenomic Organization

**Figure 4:**
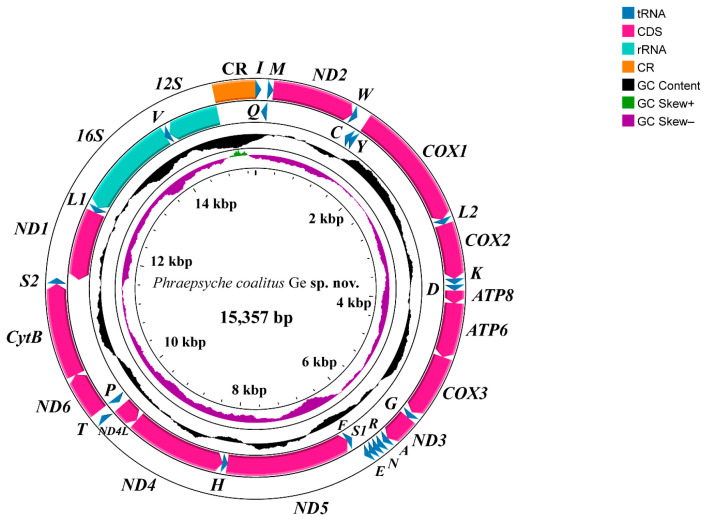
The mitogenome of *Phraepsyche coalitus* Ge **sp. nov.** is visualized as a circular map, displaying the J-strand on the outer loop and the N-strand on the inner loop.

The mitogenome of the *Phraepsyche coalitus* Ge **sp. nov.** is 15,357 bp in length and exhibits a typical circular structure (Figure 4). The mitogenome contains 37 typical genes (13 PCGs, two rRNAs, and 22 tRNAs) and one CR, which is usually present in most insect mitogenomes. The lengths of tRNA genes range from 59 bp (*trnS1*) to 71 bp (*trnI*), protein-coding genes vary from 162 bp (*ATP8*) to 1,727 bp (*ND5*), and the lengths of *16SrRNA* and *12SrRNA* are 1377 bp and 721 bp, respectively. All tRNA genes exhibit a typical cloverleaf secondary structure except for *trnS1*, which lacks the dihydrouracil arm.

The nucleotide composition of *P. coalitus* exhibits a significant AT-skew, with an overall A + T content of 80.44%. Notably, the control region (CR) showed the highest A + T content (95.56%), while site 2 of PCGs displayed the lowest A + T content (74.17%). The A + T content of tRNAs was lower than that of *16SrRNA* and *12SrRNA*. Among the protein-coding genes (PCGs), the second codon position had the lowest A + T content (74.17%), whereas the third codon position had the highest A + T content (87.86%). The mitochondrial genome of *Phraepsyche coalitus* Ge **sp. nov.** exhibited a positive AT-skew and a negative GC-skew. A negative AT-skew and GC-skew were observed in the PCGs, a negative AT-skew and a positive GC-skew were observed in the CR, and a positive AT-skew and GC-skew were detected in the tRNAs and rRNAs. Detailed nucleotide composition and skew analyses are provided in Table S4.

### 3.3. Codon Usage of PCGs

**Figure 5:**
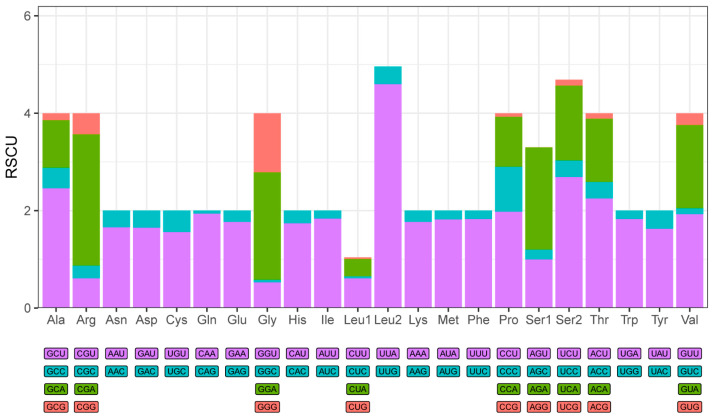
Relative synonymous codon usage (RSCU) in the mitogenome of *Phraepsyche coalitus* Ge **sp. nov.** RSCU values are defined per codon family on the *y*-axis, while codon families and their synonymous variants are grouped on the *x*-axis.

With the exception of the *COX1* gene, which started with TTG, all other protein-coding genes (PCGs) utilized typical ATN start codons. The *COX2* gene terminated with an incomplete stop codon (T), and the *ND5* gene terminated with TA, while the remaining 11 PCGs ended with the conventional TAA or TAG stop codons (Table S5). We analyzed the relative synonymous codon usage (RSCU) of the novel species. The result indicated that all 62 invertebrate codons are used in the mitogenome of *Phraepsyche coalitus* Ge **sp. nov.** High-frequency codons are UUA (458), AUU (377), and UUU (343), which are predominantly used for Leu, Ile, and Phe, respectively. The RSCU value of the AGG codon was 0 (Figure 5).

### 3.4. Phylogenetic Analyses

**Figure 6:**
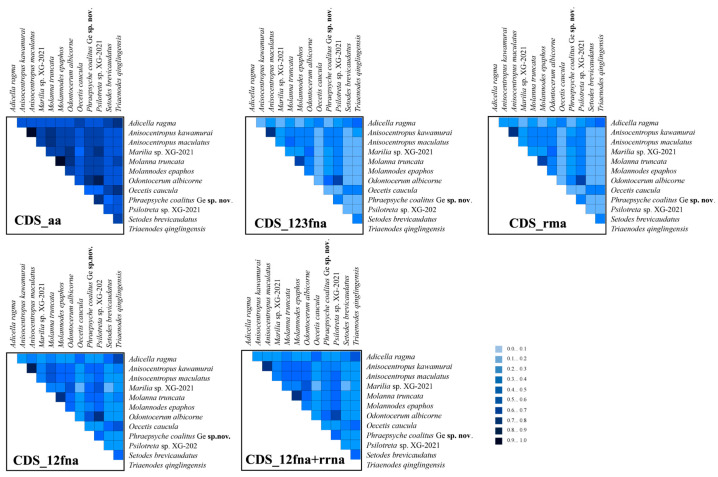
Heterogeneity of sequence composition of mitochondrial genomes for the five datasets.

The five matrices generated in this study were used to infer the phylogenetic position of the new species using maximum likelihood (ML) methods, and the results are as follows: (1) the CDS_12fna matrix contained 7288 sites; (2) the CDS_123fna matrix contained 10,932 sites; (3) the CDS_12fna+rrna matrix contained 9287 sites; (4) the CDS_rma matrix contained 12,931 sites; and (5) the CDS_aa matrix contained 3644 sites. In all datasets, the heterogeneity of sequence divergence within Odontoceridae was lower than that between Odontoceridae and species from other families (Figure 6). Among these datasets, the CDS_aa dataset exhibited the lowest sequence divergence heterogeneity, while the CDS_rrna dataset showed the highest.

**Figure 7:**
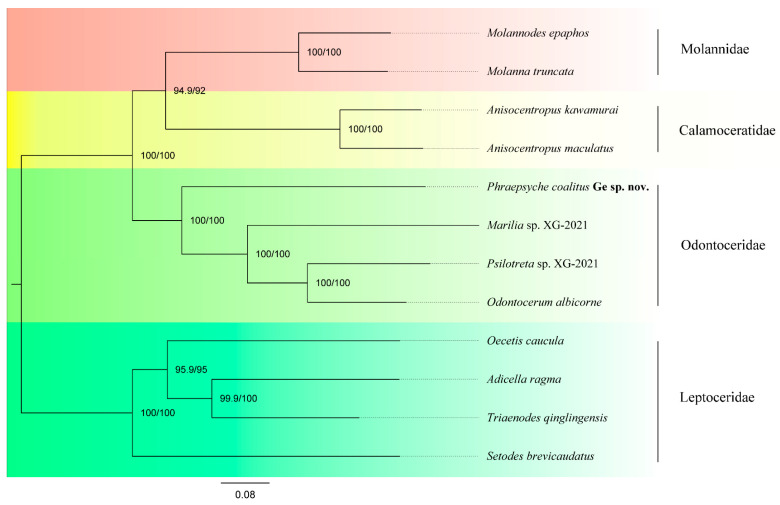
ML tree based on the dataset of CDS_aa using the mART + C60 + FO + R mixture model in IQ-tree.

Phylogenetic analyses revealed that the topological structures generated based on the five data matrices were consistent (Figure 7), and all branching nodes were well supported. The phylogenetic tree recovered the family Odontoceridae as monophyletic, and the monophyly of other families was also strongly supported (SH-aLRT/UFBoot = 100/100). Odontoceridae was sister to the clade formed by Molannidae and Calamoceratidae. Meanwhile, phylogenetic results assigned the genus *Phraepsyche* to Odontoceridae and uncovered its phylogenetic position as a basal lineage within this family. Furthermore, the mitochondrial gene rearrangement pattern detected in the genus *Marilia* was not observed in *Phraepsyche*, which further corroborated the primitive taxonomic status of *Phraepsyche*. The gene rearrangement in the genus *Marilia* may represent an apomorphy unique to this genus.

## 4. Discussion

The new species discovered in Guangxi, China, in this study represents the sixth species of the genus, substantially updating our understanding of the taxonomic diversity and geographic range of this rarely collected genus. Prior to this study, *Phraepsyche species* were previously recorded mostly from Vietnam and Thailand [5,41], with only two species known from China: *P. acuminata* from Guangxi Zhuang Autonomous Region and *P. yitungshana* from Hong Kong. The type locality of the new species lies in the China–Vietnam border region, suggesting that additional undescribed *Phraepsyche* species likely remain undiscovered in the mountainous areas of southwestern China. This discovery further highlights that our knowledge of Odontoceridae diversity in the Chinese part of the Oriental Region is still incomplete, and Guangxi, as a recognized global biodiversity hotspot, merits more intensive faunal surveys.

Morphologically, *P. coalitus* Ge **sp. nov.** can be readily distinguished from all previously described congeners by unique features of the male genitalia, most notably the divided and partially fused condition of tergum X in dorsal view, as well as the distinct length and shape of the preanal appendages and the second segment of the inferior appendages. These characters clearly separate it from the two Vietnamese species (*P. pectinata* and *P. epha*) and the Hong Kong species *P. yitungshana* [5,6,40]. Comparative morphological analysis of tergum X structures across the genus reveals two distinct evolutionary trends: gradual division and gradual fusion of the tergal plates. Unfortunately, no molecular data were previously available for *Phraepsyche*, which has hindered the reconstruction of a robust phylogenetic framework for the genus. Future discoveries of new taxa and the generation of additional molecular data will greatly improve our understanding of the origin and evolutionary history of *Phraepsyche*. In particular, phylogenomic approaches hold great promise for resolving the phylogenetic position of this enigmatic genus within Odontoceridae [29].

We present the first complete mitochondrial genome of the genus Phraepsyche, providing critical genomic resources for this rarely studied odontocerid genus. The mitogenome of *P. coalitus* Ge **sp. nov.** exhibits a typical circular molecular structure consistent with most published trichopteran mitogenomes, encoding a full set of 13 protein-coding genes (PCGs), 22 transfer RNA genes (tRNAs), two ribosomal RNA genes (rRNAs), and a 518 bp non-coding control region. Meanwhile, we reconstructed phylogenetic relationships by incorporating other families of Leptoceroidea and discussed the phylogenetic position of this genus. As no molecular data of this genus have been reported previously, we also revealed its phylogenetic placement at the mitogenome level for the first time. Notably, no gene rearrangements were detected in the *P. coalitus* Ge **sp. nov.** mitogenome, which retains the highly conserved ancestral mitochondrial gene order. Mitochondrial gene rearrangement is recognized as an important evolutionary marker in arthropods, with variable degrees of gene shuffling reported across different trichopteran lineages: derived families often exhibit lineage-specific gene rearrangements, whereas early-diverging lineages tend to preserve the ancestral genomic architecture. The complete absence of gene rearrangements in *Phraepsyche,* therefore, provides independent, genome-level evidence for its basal phylogenetic status. When combined with our phylogenetic tree topology, this highly conserved genomic architecture provides strong and congruent support that *Phraepsyche* represents an early-diverging (basal) lineage within Odontoceridae [10].

As biomonitoring studies using environmental DNA (eDNA) and metabarcoding become increasingly prevalent, the development of a robust DNA barcode reference database has become critically important [42]. In this study, we used BLAST (https://blast.ncbi.nlm.nih.gov accessed on 15 April 2026) to compare the *mtCOI* barcode sequence of *P. coalitus* Ge **sp. nov.** generated in this study against all public sequence databases, and no matching sequences from any odontocerid species were recovered. The highest sequence similarities were with *Phylloicus* sp. and *Helicopha* sp. (Table S6). These BLAST results would inevitably mislead non-taxonomist researchers conducting biodiversity surveys and aquatic biomonitoring, leading to erroneous species identifications and flawed analytical conclusions. Although DNA barcoding research on Trichoptera has made substantial progress, most efforts have been concentrated in North America, Europe, Japan, and Australia, leaving many East Asian endemic genera unrepresented in global databases [43]. As a “DNA Barcoding 2.0” resource, the mitogenome data generated in this study not only enriches the DNA barcode library for Chinese Trichoptera and provides a critical foundation for eDNA metabarcoding applications, but it also offers additional multi-locus markers for accurate species identification and larval-adult association studies.

## 5. Conclusions

In this study, we described a new species of the genus *Phraepsyche* from southwestern China, thereby enriching the species diversity of this previously understudied genus endemic to the Oriental region. More importantly, we provided the first mitochondrial genome data for *Phraepsyche*, which fills a significant gap in the genetic resources for Odontoceridae. The assembly, annotation, and structural analysis of the mitogenome, along with the phylogenetic inference based on this data, have clarified the evolutionary position of *Phraepsyche* within the family. These findings not only enhance our understanding of the biodiversity of Chinese freshwater insects but also establish a foundational genetic framework for future taxonomic and evolutionary studies of Trichoptera.

## Data Availability

Voucher specimens of the new *Phraepsyche* species and other examined specimens are deposited in the Tianjin Normal University, Tianjin, China. All specimens were collected from southwestern China with appropriate permissions. The original contributions presented in this study are included in the article. The mitochondrial genome sequences generated for the new *Phraepsyche* species will be deposited in GenBank (accession number PZ622599). Further inquiries can be directed to the corresponding author.

## Supplementary Materials

The following supporting information can be downloaded at: https://www.mdpi.com/article/10.3390/ani16142209/s1, Table S1: Distribution data of the genus *Phraepsyche*; Table S2: PCR primers used to sequence *mtCOI* genes of the new species in this study; Table S3: Detailed taxonomic information of sequences used for phylogenetic analysis in present study; Table S4: Nucleotide composition of *Phraepsyche coalitus* Ge **sp. nov.**; Table S5: Start codons and end codons of PCGs in the mitogenome of *Phraepsyche coalitus* Ge **sp. nov.**; Table S6: BLAST results of the *mtCOI* sequence of *Phraepsyche coalitus* Ge **sp. nov.** against the NCBI nucleotide database. The table summarizes the closest matches, including scientific name, accession numbers, percent identity, and query cover.
