# Supplementary material for: A New Species and Molecular Characterization of the Genus Phraepsyche Malicky and Chantaramongkol, 2000, from China (Trichoptera, Odontoceridae)
Source: Animals (Basel). 2026 Jul 16;16(14):2209. doi: 10.3390/ani16142209 (PMC13403800; doi:10.3390/ani16142209)
Supplement: Supplementary file 1 [file animals-16-02209-s001.zip › animals-4397236-supplementary.pdf]

Table S1. Distribution data of the genus *Phraepsyche*.

Species	Distribution data	Altitude	Reference
Phraepsyche danaos	Wiang Kosai Nationalpark; 17°58.00′N, 99°35.00′E	360 m	(Malicky & Sompong, 2000)
Phraepsyche epha	Vietnam, Tarn Dao; 21°28.00′N, 105°38.00′E	800-1100 m	(Malicky, 2008)
Phraepsyche pectinata	Vietnam: Lamdong: Baoloc, Duchma Stream; 11°32.88′N, 107°48.48′E	/	(Oláh & Johanson, 2010)
	Vietnam: Lamdong: Dalat, Atangla Waterfall; 11°53.93′N, 108°26.97′E	/	
Phraepsyche yitungshana	PR China: Hong Kong: Lantau South Country Park 22°15.55′N, 113°59.10′E	/	(Oláh & Johanson, 2010)
Phraepsyche acuminata	PR China: Guangxi, Tian-lin County, Cen-wang-lao-shan Provincial Forest Preserve; 24°26.54′N, 106°14.49′E	1155 m	(Yang & Morse, 2020)
	PR China: Guangxi, Long-lin County Jin-zhong-shan Provincial Forest Preserve; 24°34.95′N, 104°54.85′E	1145 m	
	PR China: Guangdong, Longmeng County Nan-kun Shan Provincial Nature Preserve; 23°38.62′N, 113°50.84′E	542 m	
Phraepsyche coalitus Ge **sp. nov.**	PR China: Guanxi, Baise City, Napo County; 23°11.85′N, 105°39.61′E,	639 m	This study
	PR China: Guanxi, Baise City, Napo County; 23°11.20′N, 105°39.77′E	487 m	

Table S2. PCR primers used to sequence *mtCOI* genes of the new species in this study.

Primer	Sequence	Reference
LCO1490	GGTCAACAAATCATAAAGATATTGG	Folmer et al., 1994
HCO2198	TAAACTTCAGGGTGACAAAAAATCA	Folmer et al., 1994

Table S3. Detailed taxonomic information of sequences used for phylogenetic analysis in present study.

Family	Genus	Species	GenBank accession number
**Ingroup**			
Calamoceratidae	*Anisocentropus*	*Anisocentropus kawamurai*	NC_069242
	Anisocentropus	*Anisocentropus maculatus*	NC_069243
Molannidae	*Molanna*	*Molanna truncata*	NC_069270
	Molannodes	*Molannodes epaphos*	NC_069271
Odontoceridae	*Marilia*	*Marilia* sp. XG-2021	OL678030
	Odontocerum	*Odontocerum albicorne*	MT483622
	Phraepsyche	*Phraepsyche coalitus* Ge **sp. nov.**	pending
	Psilotreta	*Psilotreta* sp. XG-2021	OL678046
**Outgroup**			
Leptoceridae	*Adicella*	*Adicella ragma*	NC_069240
	Oecetis	*Oecetis caucula*	NC_069274
	Setodes	*Setodes brevicaudatus*	NC_069285
	Triaenodes	*Triaenodes qinglingensis*	NC_069289

Table S4. Nucleotide composition of *Phraepsyche coalitus* Ge **sp. nov.**.

Regions	length (bp)	A(%)	T(%)	C(%)	G (%)	AG (%)	GC (%)	AT-Skew	GC-Skew
Whole genome	15,357	40.54	39.9	12.41	7.15	80.44	19.56	0.01	-0.27
PCGs	11,214	34.56	44.62	11.04	9.85	79.11	20.82	-0.13	-0.06
Site 1	3,739	36.51	37.15	10.81	15.54	73.66	26.34	-0.01	-0.18
Site 2	3,738	22.34	49.68	15.01	12.97	72.02	27.98	-0.38	0.07
Site 3	3,737	41.96	46.37	6.74	4.92	88.33	11.67	-0.05	0.16
tRNA	1,467	16.97	11.65	43.15	39.88	7.50	9.48	0.19	-0.04
l-rrna	1,377	44.3	42.92	4.21	8.57	87.22	12.78	0.02	0.34
s-rrna	721	44.8	43	4.44	7.77	87.79	12.21	0.02	0.27
CR	518	42.28	53.28	2.12	2.32	95.56	4.44	-0.12	0.05

Table S5. Start codons and end codons of PCGs in the mitogenome of *Phraepsyche coalitus* Ge **sp. nov.**.

	ATP 6	***ATP 8***	***COX 1***	***COX 2***	***COX 3***	***CYT B***	***ND1***	***ND2***	***ND3***	***ND4***	***ND4 L***	***ND5***	***ND6***
Start codon	ATG	ATT	TTG	ATT	ATG	ATG	ATA	ATT	ATA	ATA	ATG	ATA	ATA

Stop codon	TAA	TAG	TAA	T	TAA	TAA	TAA	TAA	TAA	TAA	TAA	TA	TAA

Table S6. BLAST results of the *mtCOI* sequence of *Phraepsyche coalitus* Ge **sp. nov.** against the NCBI nucleotide database. The table summarizes the closest matches, including scientific name, accession numbers, percent identity and query cover.

Scientific Name	GenBank accession number	Per.Ident	Query Cover
Phylloicus sp. INB0004363703	KX292557.1	84.40%	96.00%
Helicopha loripes	EF395015.1	84.20%	96.00%
Helicopha loripes	EF395017.1	84.20%	96.00%
Helicopha einap	EF395034.1	84.17%	96.00%
Phylloicus sp. INB0004363699	KX292518.1	84.15%	96.00%
Helicopha einap	EF395029.1	84.07%	96.00%
Helicopha paniensis	EF395010.1	84.02%	96.00%
Helicopha einap	EF395036.1	83.92%	96.00%
Phylloicus elegans	HQ967629.1	83.84%	96.00%
Phylloicus holzenthali	HQ967578.1	83.71%	96.00%
Phylloicus ephippium	HQ967632.1	83.61%	96.00%
